# Neuroblastoma cells depend on HDAC11 for mitotic cell cycle progression and survival

**DOI:** 10.1038/cddis.2017.49

**Published:** 2017-03-02

**Authors:** Theresa M Thole, Marco Lodrini, Johannes Fabian, Jasmin Wuenschel, Sebastian Pfeil, Thomas Hielscher, Annette Kopp-Schneider, Ulrike Heinicke, Simone Fulda, Olaf Witt, Angelika Eggert, Matthias Fischer, Hedwig E Deubzer

**Affiliations:** 1 Department of Pediatric Hematology, Oncology and SCT, Charité–Universitätsmedizin Berlin, Campus Virchow-Klinikum, Augustenburger Platz 1, Berlin 13353, Germany; 2 Department of Pediatric Hematology and Oncology, University of Heidelberg, INF 430, Heidelberg 69120, Germany; 3 Clinical Cooperation Unit Pediatric Oncology, German Cancer Research Center (DKFZ) and German Consortium for Translational Cancer Research (DKTK), INF 280, Heidelberg 69120, Germany; 4 Department of Biostatistics, German Cancer Research Center (DKFZ), INF581, Heidelberg 69120, Germany; 5 Institute for Experimental Cancer Research in Pediatrics, J. W. Goethe University Hospital Frankfurt, Korntur Str. 3a, Frankfurt am Main 60528, Germany; 6 German Cancer Consortium (DKTK), INF 280, Heidelberg 69120, Germany; 7 German Cancer Research Center (DKFZ), INF 280, Heidelberg 69120, Germany; 8 Department of Pediatric Hematology and Oncology, University of Cologne, Kerpener Str. 62, Cologne 50937, Germany; 9 Department of Children and Adolescent Medicine, Center for Molecular Medicine Cologne, University of Cologne, Robert-Koch-Str. 21, Cologne 50931, Germany; 10 Junior Neuroblastoma Research Group, Experimental and Clinical Research Center (ECRC) of the Max-Delbrück Center for Molecular Medicine (MDC) in the Helmholtz Community and the Charité–University Medicine Berlin, Lindenberger Weg 80, Berlin 13125, Germany

## Abstract

The number of long-term survivors of high-risk neuroblastoma remains discouraging, with 10-year survival as low as 20%, despite decades of considerable international efforts to improve outcome. Major obstacles remain and include managing resistance to induction therapy, which causes tumor progression and early death in high-risk patients, and managing chemotherapy-resistant relapses, which can occur years after the initial diagnosis. Identifying and validating novel therapeutic targets is essential to improve treatment. Delineating and deciphering specific functions of single histone deacetylases in neuroblastoma may support development of targeted acetylome-modifying therapeutics for patients with molecularly defined high-risk neuroblastoma profiles. We show here that HDAC11 depletion in *MYCN*-driven neuroblastoma cell lines strongly induces cell death, mostly mediated by apoptotic programs. Genes necessary for mitotic cell cycle progression and cell division were most prominently enriched in at least two of three time points in whole-genome expression data combined from two cell systems, and all nine genes in these functional categories were strongly repressed, including *CENPA*, *KIF14*, *KIF23* and *RACGAP1*. Enforced expression of one selected candidate, *RACGAP1*, partially rescued the induction of apoptosis caused by HDAC11 depletion. High-level expression of all nine genes in primary neuroblastomas significantly correlated with unfavorable overall and event-free survival in patients, suggesting a role in mediating the more aggressive biological and clinical phenotype of these tumors. Our study identified a group of cell cycle-promoting genes regulated by HDAC11, being both predictors of unfavorable patient outcome and essential for tumor cell viability. The data indicate a significant role of HDAC11 for mitotic cell cycle progression and survival of *MYCN*-amplified neuroblastoma cells, and suggests that HDAC11 could be a valuable drug target.

Neuroblastoma, a neuroectodermally derived embryonic tumor and most common extracranial tumor of childhood, remains a major cause of cancer-related deaths in children, mostly due to systemic and resistant relapses.^1^ *MYCN* oncogene amplifications,^2, 3^ *TERT* activation by genomic rearrangements,^4, 5^ *ATRX* loss-of-function mutations/deletions^6^ and germline/somatic activating *ALK* mutations^7, 8, 9, 10^ define patient subgroups at high risk for failing primary long-term remission despite aggressive multimodal treatment. Treatment for relapsed neuroblastoma and even first-line therapy for molecularly defined high-risk disease is currently undergoing a paradigm shift from classical cytotoxic agent combinations toward incorporating targeted drugs and immunotherapeutics into treatment schedules. Developing how and when to combine these newer precise therapeutics with established treatment elements such as classical chemotherapy is challenging.

Drugs currently under clinical investigation for relapsed/refractory neuroblastoma patients include the mTOR inhibitor, rapamycin (NCT01467986), and the ALK inhibitors, crizotinib (NCT00939770, NCT02559778, NCT01606878, NCT02034981) and LDK378 (NCT01742286). The strong anti-tumoral activities of inhibitors for HDAC family members, AURKA, BET/bromodomain-containing proteins, MDM2, MAP2K1 (formerly MEK) and CDK4/6 observed in preclinical neuroblastoma models^11, 12, 13, 14, 15, 16^ suggest that further drugs will enter pediatric phase I testing in the near future. Inhibitors of class I or all classical histone deacetylases that are currently approved or being evaluated in clinical cancer trials in adults^17^ give rise to mostly hematological dose-limiting toxicities, presumably due to the inhibition of several family members. This could potentially be overcome by selective blockade of single HDAC family members.

Further advances in understanding the role of single histone deacetylases are a prerequisite to fully exploiting the level of plasticity that can be therapeutically addressed with this class of acetylome-modifying drugs. MYCN was shown to recruit HDAC1/2/3/5 to promoter sites to repress transcription in neuroblastoma cells,^18, 19, 20, 21^ whereas HDAC8 and HDAC10 inhibit differentiation and promote autophagy-mediated survival in a *MYCN*-independent manner.^22, 23^ Gao *et al.*^24^ identified and characterized *HDAC11* in 2002, the only class IV HDAC family member identified to date, which is located within the ~25 kb region of chromosome 3p25.1. Expression of the FLAG-tagged 347 amino acid open reading frame in human embryonic kidney 293 cells, demonstrated the protein primarily localizes to the nucleus and is capable of deacetylating a synthetic peptide derived from histone H4.^24^ Subsequent studies in recent years have shed light on the role of HDAC11 in health and disease.^25^ Here we aimed to assess the significance of HDAC11 expression for neuroblastoma biology, which is as yet unknown. We recently showed that HDAC11 plays an important role in controlling proliferation in several carcinoma cell lines.^26^ BE(2)-C and IMR-32 were chosen as representative cell lines for the high-risk neuroblastoma subtype characterized by *MYCN* amplification and loss of heterozygosity at chromosome 1p. The BE(2)-C cell line, established from a bone marrow metastasis after the patient had received 5 months of polychemotherapy,^27^ is hemizygous for *TP53*, with the remaining allele harboring a missense mutation at codon 135 that renders TP53 non-function and the cell line resistance to doxorubicin.^28^ IMR-32 cells harbor a partial *ALK* amplification, resulting in constitutive ALK activity leading to uncontrolled proliferation.^29^ We assessed the effect of HDAC11 depletion on phenotype in these two neuroblastoma cell models and performed whole-genome expression profiling to decipher the pathways triggering the HDAC11 depletion phenotype in neuroblastoma cells.

## Results

### HDAC11 depletion in neuroblastoma cells triggers cell death preceded by aberrant mitotic spindle assemblies

**Figure 1:**
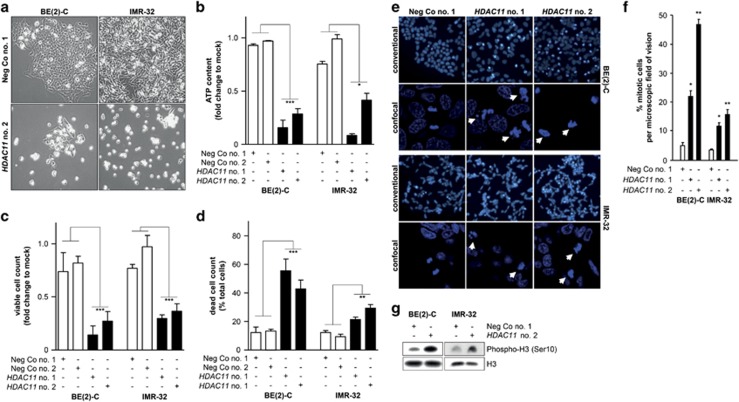
HDAC11 depletion in neuroblastoma cells causes aberrant mitosis and cell death. (**a**) Phase contrast microscopy pictures showing the phenotype of BE(2)-C and IMR-32 neuroblastoma cells 96 h after transfection with *HDAC11*- or negative control siRNA. Original magnification × 200. (**b**) Intracellular ATP content of BE(2)-C and IMR-32 cultures 96 h after transfection with *HDAC11*- or negative control siRNAs (mean fold change over mock-transfected cells±S.D. is shown, *n*=3). (**c** and **d**) Viable and dead BE(2)-C and IMR-32 cell count using a semi-automatic VI-CELL Cell Viability Analyzer 96 h after transfection with *HDAC11*- or negative control siRNAs. Mean fold change over viable mock-transfected cells±S.D. is shown in (**c**), *n*⩾3. Mean percentage of dead cells is shown in **d**, *n*⩾3. (**e**) Fluorescent microscopic and confocal images of DAPI-stained DNA in BE(2)-C and IMR-32 cells transfected with *HDAC11*- or negative control siRNA for 48 h. DAPI-stained cells were analyzed using the × 20 objective on a conventional widefield microscope and the × 63 objective on a confocal microscope. Arrowheads indicate aberrantly constituted mitotic spindle assemblies. (**f**) Quantification of the fluorescent microscopic images shown in **e**. At least 10 microscopic fields were evaluated per treatment group by two experimenters. (**g**) Western blot analysis of phospho Histone H3 (Ser10) expression 48 h after transfection of *HDAC11*- or negative control siRNA. Histone H3 served as loading control. *P<0.05; **P⩽0.01; ***P⩽0.001

To decipher mechanisms controlled by HDAC11 in neuroblastoma cells, we assessed phenotypic appearance, intracellular adenosine triphosphate (ATP) content, and number of viable and dead cells following transient HDAC11 knockdown in BE(2)-C and IMR-32 cells. Two different siRNAs directed against *HDAC11* (Supplementary Table S1) were used to control unspecific and off-target effects. HDAC11 expression was reduced up to 95% on the mRNA level, as measured by qRT-PCR (Supplementary Table S2; Supplementary Figures S1a–b), and up to 85% on the protein level related by western blotting (Supplementary Figures S1c–d). Cell culture density was reduced by HDAC11 depletion, and detached single cells and clusters floated in the culture medium 96 h after transfection (Figure 1a), suggesting proliferative inhibition and cell death induction. Intracellular ATP content was quantified 96 h after transfection by the CellTiter-Glo assay. HDAC11 depletion diminished ATP content in BE(2)-C and IMR-32 cells by 59–93%, compared to cells transfected with negative controls (Figure 1b), indicating that loss of HDAC11 activity reduced metabolic activity. Consistent with this finding, semi-automated cell viability analysis based on trypan blue exclusion revealed a 63–86% reduction in the number of membrane-intact viable BE(2)-C or IMR-32 cells 96 h after HDAC11 depletion (Figure 1c), whereas the number of membrane-permeable dead cells increased by 1.8- to 4.5-fold (Figure 1d). Transient HDAC11 knockdown in the SH-SY5Y and SK-N-AS neuroblastoma cell lines, which lack *MYCN* amplification, diminished ATP content by 21–38% (Supplementary Figures S2a–b). Semi-automated analyses detected a 32–45% reduction in the number of viable SH-SY5Y or SK-N-AS cells 96 h after HDAC11 depletion and a 1.3- to 1.7-fold increase in the number of dead cells (Supplementary Figures S2c–d). We observed an apparent increase in mitotic BE(2)-C and IMR-32 cells using fluorescent microscopy of diamidino-2-phenylindole (DAPI)-stained adherently growing cells 48 h after HDAC11 depletion and prior to time points assessing cell death (Figure 1e). Quantifying the percentage of mitotic cells per microscopic field showed HDAC11 depletion increased mitotic BE(2)-C cells from 5% to 22–47% and mitotic IMR-32 cells from 4% to 12–16% (Figure 1f). Confocal microscopy of the DAPI-stained cells revealed aberrantly constituted mitotic spindle assemblies in HDAC11-depleted cells while negative controls harbored normal appearing mitotic spindle assemblies (Figure 1e). Ser10 of histone H3 is phosphorylated in mitotic cells, allowing direct quantification of mitotic cells. HDAC11 depletion increased phospho H3 (Ser10) levels by approximately three-fold in BE(2)-C and IMR-32 cells 48 h after siRNA transfection (Figure 1g), supporting the increase in mitotic cells observed microscopically. Fluorescence-activated cell sorting (FACS) of propidium iodide-stained BE(2)-C cells demonstrated an increase of cells in G2/M from 33 to 51% 48 h after HDAC11 depletion (Supplementary Figures S3a–e). Our results in neuroblastoma cell lines with and without *MYCN* amplifications collectively highlight the significance of HDAC11 expression for cellular viability, particularly in the presence of *MYCN* amplification. Taken together, our experiments show that HDAC11 depletion in neuroblastoma cells causes formation of aberrant mitotic spindle assemblies followed by increased cell death.

### HDAC11 depletion in neuroblastoma cells induces apoptosis

**Figure 2:**
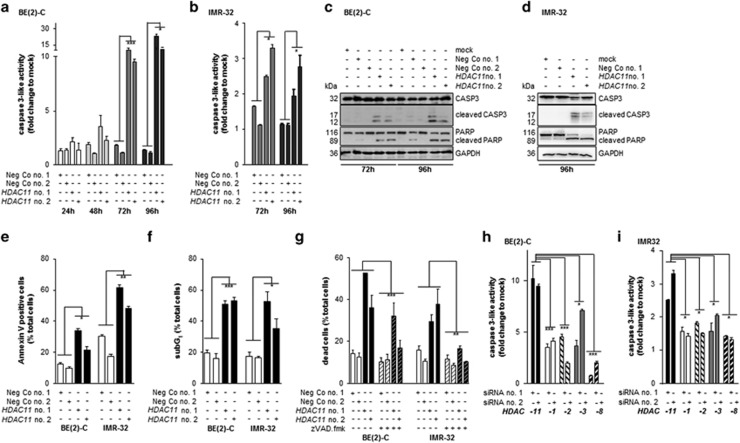
HDAC11 depletion in neuroblastoma cells triggers caspase activation and caspase-dependent apoptosis. (**a** and **b**) Caspase 3-like activity was measured in a caspase 3-like activity assay in time course 24–96 h after transfection of BE(2)-C and 72–96 h after transfection of IMR-32 cells with *HDAC11*- or negative control siRNAs (mean fold changes over mock±S.D., *n*=3). (**c** and **d**) CASP3 and PARP cleavage was analyzed by western blotting 72–96 h after transfection of BE(2)-C (**c**) and IMR-32 cells (**d**) with *HDAC11*- or negative control siRNAs. Shown are the full-length CASP3 (32 kDa) and poly (ADP-ribose) polymerase (PARP) proteins (116 kDa), and the active cleavage bands of CASP3 (17, 12 kDa) and PARP (89 kDa). GAPDH served as a loading control. (**e** and **f**) Early- and late-stage apoptosis was determined by staining of annexin V shifted to the outer plasma membrane 72 h after siRNA transfection (**e**; mean±S.D., *n*⩾5) and by quantifying DNA fragmentation in propidium iodide-stained nuclei at 96 h (**f**; mean±S.D., *n*=3). (**g**) BE(2)-C and IMR-32 cells were transfected with *HDAC11*- or negative control siRNAs and treated with 20 *μ*M zVAD.fmk or solvent control for 96 h. Shown is the mean percentage of dead cells±S.D. measured with a semi-automated VI-CELL Cell Viability Analyzer (*n*⩾3). (**h** and **i**) Comparison of caspase 3-like activity in BE(2)-C (**h**) and IMR-32 cells (**i**) transfected for 72 h with *HDAC11* siRNAs or siRNAs directed against the class I HDACs 1, 2, 3 or 8 (mean fold change over mock±S.D., *n*=3). *P<0.05; **P⩽0.01; ***P⩽0.001

To test whether apoptosis was involved in the phenotype observed, we conducted caspase 3-like activity assays, PARP western blotting, quantification of cell surface phosphatidylserine expression and DNA fragmentation by FACS. The time–response kinetic investigating caspase 3-like activity 24–96 h after siRNA transfection in BE(2)-C cells demonstrated that HDAC11 depletion elicited an ~10-fold increase in caspase 3-like activity over negative controls at 72 h, reaching 11- to 23-fold at 96 h (Figure 2a). Caspase 3-like activity in IMR-32 was elevated up to 3.5-fold above negative controls (Figure 2b). Caspase 3 cleavage fragments were detected in BE(2)-C and IMR-32 cells by western blotting (Figures 2c and d). Caspase-mediated apoptosis involves cleavage of numerous substrates including PARP1, a nuclear enzyme involved in DNA repair. Western blotting detected increased cleavage to the 89-kDa PARP1 fragment 72 and 96 h after HDAC11 depletion (Figures 2c and d). To collect further evidence that HDAC11 depletion induces apoptosis, we analyzed the shift of phosphatidylserine from the inner to the outer plasma membrane leaflet during apoptosis using annexin V staining. Outer surface localization of phosphatidylserine increased by 1.6- to 3.5-fold in BE(2)-C and IMR-32 cells 72 h after siRNA transfection for HDAC11 depletion (Figure 2e; Supplementary Figure S4a). The percentage of cells with fragmented DNA (sub-G1 fraction) was determined by FACS analysis of propidium iodide-stained cells. HDAC11 depletion triggered a 2- to 3.2-fold increase in sub-G1 BE(2)-C and IMR-32 cells, reaching up to 62% 96 h after transfection (Figure 2f; Supplementary Figure S4b). To assess the contribution of apoptosis to the phenotype mediated by HDAC11 depletion, we combined treatment with the pan-caspase inhibitor, zVAD.fmk, with transfection of *HDAC11*-directed or negative control siRNA, then quantified the number of dead BE(2)-C and IMR-32 cells by semi-automated trypan blue staining. Stable inhibition of proteolysis by activated caspases substantially prevented cell death induction by HDAC11 depletion in BE(2)-C and IMR-32 cells (Figure 2g). The dependence of cell death induction on caspase activity, the confirmation of PARP1 cleavage, the increased cell surface localization of phosphatidylserine and the presence of DNA fragmentation in dead or dying cells strongly support a major involvement of the apoptotic machinery in the phenotype observed.

We compared the induction of caspase 3-like activity by HDAC11 depletion in BE(2)-C cells with that achieved by singly depleting HDAC1, HDAC2, HDAC3 or HDAC8 (Supplementary Figures 1e–h). While HDAC11 depletion resulted in an ~10-fold increase in caspase 3-like activity 72 h after transfection (compare Figure 2a with Figure 2h), caspase 3-like activity in BE(2)-C cells depleted for HDAC1, HDAC2 or HDAC8 was below five-fold of the respective controls and below 7.5-fold of controls for HDAC3-depleted cells (Figure 2h). Likewise, HDAC11 depletion induced caspase 3-like activity in IMR-32 cells more strongly than singly depleting HDAC1, HDAC2, HDAC3 or HDAC8 (Figure 2i). The strength of induction of caspase 3-like activity by HDAC11 depletion suggests that HDAC11 could be a valuable drug target for selective inhibition in concert with other targeted therapeutics.

### HDAC11 influences genes involved in mitotic cell cycle progression and cell division

**Figure 3:**
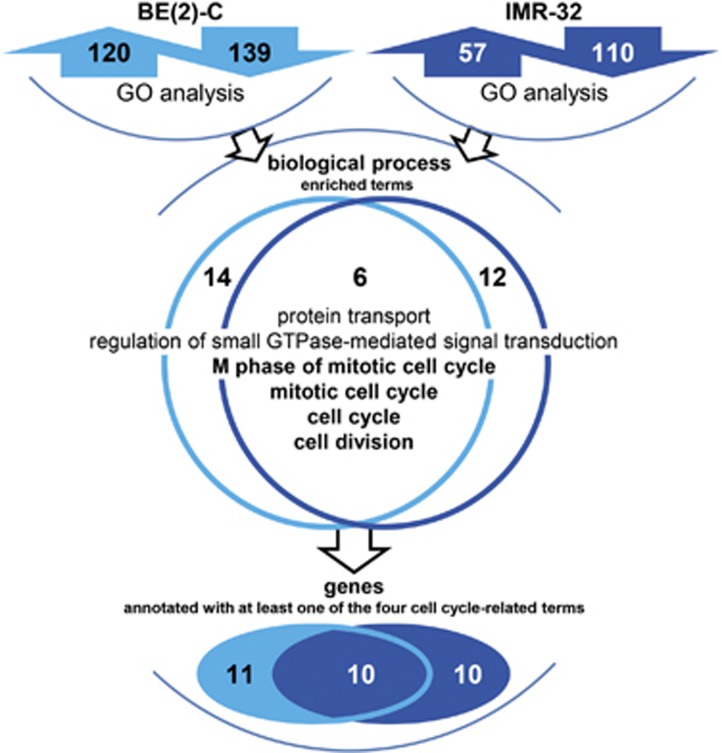
Schematic model showing an enrichment of biological function terms associated with cell cycle and cell division in a GO analysis of whole-genome gene expression data obtained in time course (42, 48, 54 h) from the BE(2)-C and IMR-32 cell systems transfected with negative control siRNA or two different *HDAC11*-specific siRNAs

**Figure 4:**
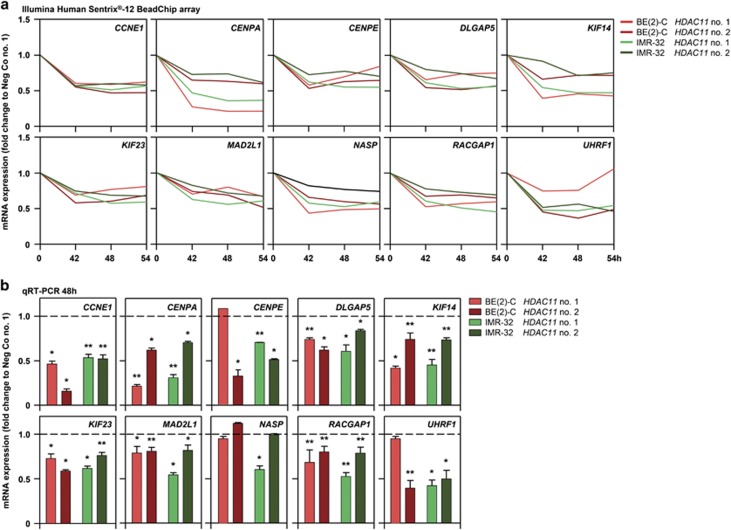
Expression of a group of genes related to the cell cycle and cell division following HDAC11 depletion in neuroblastoma cells. (**a**) Time course of mRNA expression of differentially regulated genes associated with the GO terms M-phase of mitotic cell cycle, mitotic cell cycle, cell cycle or cell division. BE(2)-C and IMR-32 cells were transfected with negative control siRNA or two different *HDAC11*-specific siRNAs for whole-genome expression analysis (42, 48, 54 h, *n*=2) Mean fold change over negative control siRNA is shown. (**b**) Validation experiments using qRT-PCR are shown for the 48 h time point (mean fold change over negative control siRNA±S.D., *n*⩾3). *P<0.05; **P⩽0.01

To detect transcriptomic changes by HDAC11 depletion in a time-resolved manner, whole-genome expression profiles of BE(2)-C and IMR-32 cells were generated using Illumina Human Sentrix-12 BeadChip arrays 42, 48 and 54 h after transfection with the two different siRNAs targeting *HDAC11* or a negative control (compare with Figures 1,2). In total, 259 genes were regulated (120 up and 139 down) by HDAC11 depletion in BE(2)-C, and 167 genes were regulated (57 up and 110 down) by HDAC11 depletion in IMR-32 cells (Supplementary Information). Genes differentially expressed relative to controls at two or more time points in both cell lines (*P*<0.05) after HDAC11 depletion were included in gene ontology (GO) analyses. This approach identified 20 enriched biological process terms in BE(2)-C cells and 18 in IMR-32 cells, of which six overlapped in both cell models (Figure 3). Biological processes enriched in both cell lines included M-phase of mitotic cell cycle, mitotic cell cycle, cell cycle, cell division, protein transport and small GTPase-mediated signal transduction. Finding four biological function terms related to cell cycle and mitosis in the unbiased analysis of expression in time course from both cell lines coupled with our detecting cells accumulating in mitosis then undergoing apoptosis following HDAC11 depletion prompted us to focus on genes related to the terms, cell cycle and mitosis. In total, 21 and 20 genes representing these terms were regulated in BE(2)-C and IMR-32 cells, respectively (Figure 3), with 10 genes being regulated in both cell lines (Figures 3,4a). Downregulation was confirmed for 9 (*CCNE1*, *CENPA*, *CENPE*, *DLGAP5*, *KIF14*, *KIF23*, *MAD2L1*, *RACGAP1* and *UHRF1*) of the 10 genes using qRT-PCR (Figure 4b, exemplarily shown for 48 h). Taken together, the HDAC11 depletion phenotype characterized by cell accumulation in mitosis and aberrant spindle assembly formation is associated with downregulation of genes required for mitotic cell cycle progression in neuroblastoma cells.

We next turned to two independently existing whole-genome expression profiles from 476^30^ and 88^6^ primary neuroblastomas to investigate the correlation between overall and event-free survival in patients with tumors expressing *CCNE1*, *CENPA*, *CENPE*, *DLGAP5*, *KIF14*, *KIF23*, *MAD2L1*, *RACGAP1* or *UHRF1*. High-level expression of each single gene strongly correlated with unfavorable overall and event-free patient survival in the entire cohort and in the patient subgroup with tumors lacking *MYCN* amplifications (Table 1; Supplementary Table S3). No major differences in the expression of these genes were observed in the patient subgroup with tumors harboring *MYCN* amplifications (data not shown). High-level expression of each single gene also strongly correlated with the expression of each of the other seven genes except *CCNE1*, supporting the concept of a functionally linked gene group and suggesting a similar pattern of regulation (Supplementary Figure S5, exemplarily shown for the Oberthuer cohort^30^). We also assessed expression of the nine genes in high-risk neuroblastomas (as defined by INRG staging^31^) with (*n*=67) and without (*n*=77) *MYCN* amplifications in the Kocak tumor cohort.^30^ High-level expression of the 9-gene group was correlated (*P*=2.08^e^^−08^) with *MYCN* amplification. Neuroblastomas with unfavorable biology and associated dismal patient outcome strongly express this 9-gene group associated with cell cycle progression.

### Counteracting RACGAP1 downregulation partially rescues caspase 3 induction by HDAC11 depletion

**Figure 5:**
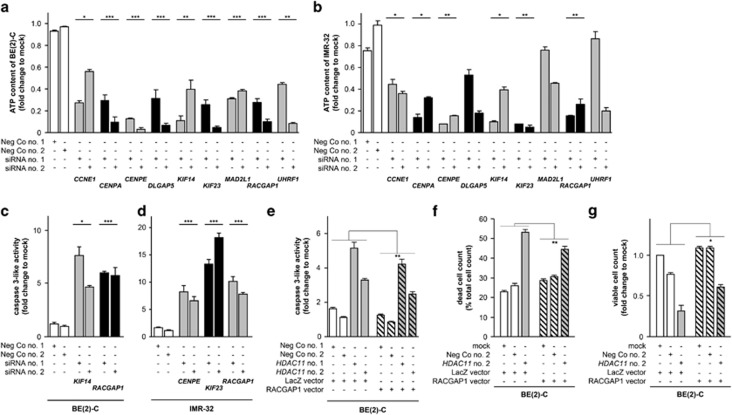
Enforced RACGAP1 expression partially rescues apoptosis triggered by HDAC11 depletion. (**a** and **b**) Intracellular ATP content of BE(2)-C (**a**) and IMR-32 cultures (**b**) 96 h after transfection with candidate gene-specific or negative control siRNAs (mean fold change over mock-transfected cells±S.D. is shown, *n*=3). (**c** and **d)** Caspase 3-like activity of BE(2)-C (**c**) and IMR-32 cultures (**d**) 72 h after transfection with candidate gene-specific or negative control siRNAs (mean fold changes over mock±S.D., *n*=3). (**e**–**g**) Caspase 3-like activity (**e**), dead cell count (**f**) and viable cell count (**g**) of BE(2)-C cells 72 h after transfection with *HDAC11*-specific or negative control siRNAs and 48 h after transfection of the RACGAP1 plasmid or respective *LacZ* control (mean fold changes over mock±S.D., *n*=3). *P<0.05; **P⩽0.01; ***P⩽0.001

We aimed to test whether enforced expression of one of the 9-gene group downregulated by HDAC11 depletion should partially rescue the cell death phenotype. Both a medium-throughput CellTiter-Glo assay measuring ATP content and a medium-throughput CaspGlo assay were performed in BE(2)-C and IMR-32 cells after depletion of each of the nine genes to identify the most suitable for the rescue experiment. RNAi targeting the nine genes, each with two different siRNAs, reduced ATP content in BE(2)-C cells by at least 40% and up to 95% 96 h after transfection (Figure 5a). ATP content in IMR-32 cells was diminished by at least 55% and up to 95% after knockdown of *CCNE1*, *CENPA*, *CENPE*, *KIF14*, *KIF23* or *RACGAP1*, whereas targeting *DLGAP5*, *MAD2L1* or *UHRF1* did not largely shift ATP content (Figure 5b). Caspase 3-like induction was assessed in cells 72 h after knockdown of those genes which significantly influenced cellular ATP in both cell lines. In BE(2)-C cells, *KIF14* or *RACGAP1* knockdown caused a 4.5- to 7.7-fold induction of caspase 3-like activity (Figure 5c). In IMR-32 cells, caspase 3-like activity was triggered 6.5- to 18.2-fold by siRNA-mediated knockdown of *CENPE*, *KIF23* or *RACGAP1* (Figure 5d), and *RACGAP1* was identified as the most suitable gene for rescue experiments. Plasmid-mediated, enforced *RACGAP1* expression to counteract *RACGAP1* downregulation by HDAC11 depletion (Supplementary Figures 6a–c) significantly reduced (*P*<0.01) induction of caspase 3-like activity, decreased the number of membrane-permeable dead BE(2)-C cells and enhanced the number of viable BE(2)-C cells compared to HDAC11-depleted BE(2)-C cells transfected with the LacZ expression plasmid (Figures 5e–g), demonstrating the involvement of *RACGAP1* downregulation in the HDAC11 depletion phenotype.

## Discussion

Our studies unravel a critical role for HDAC11 in cell cycle progression and viability of *MYCN*-amplified neuroblastoma cells. We recently reported that *HDAC11* transcript levels are significantly higher in several carcinoma entities than corresponding healthy tissues.^26^ HDAC11 depletion was sufficient to inhibit metabolic activity and induce cell death in carcinoma cell lines while having no detectable effects on two different normal cell types, making HDAC11-inhibiting drugs highly interesting for programs searching for new targeted therapeutics to treat breast, colon, ovary and prostate cancers.^26^ These results suggest tumor selectivity and a relatively broad therapeutic window, which is important for the future development of small molecules selectively inhibiting HDAC11. Here we deepen the understanding of HDAC11 function in neuroblastoma. Broad-spectrum HDAC inhibitors have different effects on the cell cycle of transformed cells, including cell cycle arrests in both G1 and G2/M.^32^ As transformed cells frequently lack a functional G2 checkpoint, cancer cells arrested in G2/M frequently undergo apoptosis.^32^ Our observation that HDAC11-depleted neuroblastoma cells accumulate in G2/M, form aberrant spindle assemblies and subsequently undergo apoptosis suggest the effect elicited by pan-HDAC inhibition is at least in part mediated by targeting HDAC11.

On a molecular level, downregulation of a 9-gene group required for proper mitotic cell cycle progression was observed in an unbiased transcriptome-wide approach. Knockdown of these genes (*CCNE1*, *CENPA*, *CENPE*, *DLGAP5*, *KIF14*, *KIF23*, *MAD2L1*, *RACGAP1* and *UHRF1*) by siRNAs recapitulated the HDAC11 depletion phenotype, underlining their importance for mitosis. Counteracting downregulation of one of these genes, *RACGAP1*, partially rescued the programmed cell death phenotype observed. This supports the functional relevance of *RACGAP1* downregulation for the HDAC11 depletion phenotype. High-level mRNA expression of the nine genes correlated with unfavorable patient survival in two independent neuroblastoma cohorts as well as with *MYCN* amplification in primary high-risk neuroblastomas. Targeting rapid proliferation in *MYCN-*amplified neuroblastoma cells by downregulating genes indispensable for cell cycle progression has previously been shown to elicit cell cycle arrest and cell death, and is considered a promising strategy to block the oncogenic effect of *MYCN* amplification on cell cycle.^33^ The strong phenotype produced by HDAC11 depletion in *MYCN*-amplified neuroblastoma cell lines supports this approach.

A review of the literature solidifies the importance of the nine genes downregulated by HDAC11 depletion for proper cell cycle progression and division, but also reveals associations with other hallmarks of cancer. Enforced MYCN expression in SH-EP cells triggers transcriptional activation of cyclin E1 (*CCNE1*), and high-level *CCNE1* expression correlates with *MYCN* amplification in primary neuroblastomas.^34^ Ubiquitin like with PHD and ring finger domains 1 (UHRF1) encodes a member of a subfamily of RING finger type E3 ubiquitin ligases that is required for inheriting methylation during S-phase.^35^ Centromere protein A (CENPA) is a histone H3 variant that epigenetically marks where a centromere will form, and creates a unique more open nucleosome structure allowing recruitment of CENPC and centrosome establishment.^36^ In fact, CENPC alone can trigger kinetochore assembly in defined templates *in vitro*.^37^ Although CENPA and CENPC are parts of the inner kinetochore,^38, 39^ centromere protein E (CENPE) is a kinesin-like motor protein strongly expressed in G2 and essential for chromosome alignment by connecting the outer kinetochore to the spindle microtubule.^40^ Several studies have established that CENPE knockdown causes G2 blockade, although cell fate following the G2 block appears to rely on other genes expressed in the cell.^41^ Serial transcriptomic analyses of pre-neoplastic ganglia and end-stage tumors in the TH-MYCN transgenic mouse model identified increased *CENPE* expression during tumor progression, and targeting CENPE with the GSK923295 small-molecule inhibitor reduced proliferation in human neuroblastoma cell lines and tumor growth in three xenograft models.^42^ DLG-associated protein 5 (DLGAP5) is part of a multicomponent complex and associates with microtubules, thus, mediating stabilization.^43^ Kinesin family member 14 (KIF14) encodes a member of the kinesin-3 superfamily of microtubule motor proteins that acts to bundle and stabilize midbody microtubules during cytokinesis.^44^ *KIF14* acts as an oncogene in many cancer entities, including esophageal squamous cell carcinoma,^45^ cervical and ovarian cancers,^46, 47^ gliomas,^48^ retinoblastoma,^49, 50^ glioblastoma,^51^ hepatocellular carcinoma,^52^ lung adenocarcinoma,^53^ laryngeal carcinoma,^54^ synovial carcinomas^55^ and papillary renal cell tumors.^56^ *KIF14* is present in the minimal region of chromosome 1q gain in breast cancer cell lines, is overexpressed in breast cancers and its expression positively correlates with tumor aggressiveness.^57, 58^ KIF14 not only drives cell proliferation, but has been shown to promote chemoresistance via AKT signaling in triple-negative breast cancer^59^ and enhance metastatic and invasive capacity during breast cancer progression via RAP1A-RADIL signaling inhibition.^60^ *KIF14* is also a key gene necessary for perineural invasion by pancreatic carcinoma.^61^ High-level *KIF14* expression has also been reported in cell lines derived from medulloblastomas.^62^ Together with the results we report here, these data show that *KIF14* hyperactivity resulting from direct genomic gain or gene upregulation via epigenetic or other mechanisms is associated with the most aggressive subgroups of not only adult cancers but most pediatric embryonal tumors. It is interesting to note that *KIF14* expression is associated with the more aggressive tumor types of all carcinoma types reported to be sensitive to HDAC11 inhibition.^26^ The MAD2 mitotic arrest deficient-like 1 protein (MAD2L1) is a component of the mitotic spindle assembly checkpoint that delays anaphase onset until all chromosomes are properly aligned at the metaphase plate.^63^ *KIF23* is part of the 157-gene signature for *MYCN* activity identified by shRNA-mediated *MYCN* silencing in neuroblastoma cells and confirmed by expression studies in 88 neuroblastomas.^64^ KIF23 and Rac GTPase activating protein 1 (RACGAP1) are highly expressed during G2/M.^65, 66^ RACGAP1 and KIF23 form the centralspindlin complex,^67^ a motor complex essential for virtually every step in cytokinesis including mitotic spindle formation and anchoring. Absence of either protein leads to failure of cytokinesis due to deficient central spindle assembly and contractile ring formation.^68^ The aberrant spindle assembly formation we observed in HDAC11-depleted neuroblastoma cells suggests that HDAC11 disrupts the complex machinery regulating these processes, likely via *KIF23*, *MAD2L1* and *RACGAP1*. High-level *RACGAP1* expression was also observed in different cancer entities.^69, 70, 71, 72^ The nine genes we show downregulated by HDAC11 depletion in G2/M-arrested neuroblastoma cells all play an important role in proper cell cycle progression and division, but some are also key players in cancer hallmarks associated with progression to more aggressive tumor phenotypes, including development of chemoresistance, metastases and invasive potential. HDAC11 may provide a druggable regulatory node to reduce many functions necessary for developing aggressiveness in multiple cancers affecting adults and children.

Our previous investigations unraveled specific and non-redundant oncogenic functions of HDAC2/3/5/8/10 in neuroblastoma pathophysiology.^19, 20, 23, 73, 74^ The clinical application of pan-HDAC inhibitors showed dose-limiting toxicities, which is not surprising considering the central role of HDACs in modulating chromatin structure and cytoplasmic processes such as autophagy. Selective inhibitors may improve anti-tumoral efficacy, and have already been successfully developed against single HDACs. HDAC11-selective inhibitors have as yet not been developed. Here we show that HDAC11 depletion in *MYCN-*amplified neuroblastoma cells triggers programmed cell death preceded by an accumulation of mitotic cells characterized by aberrant spindle assembly formation. On a molecular level, downregulation of a 9-gene group required for proper mitotic cell cycle progression was observed in an unbiased transcriptome-wide approach, and counteracting *RACGAP1* downregulation by HDAC11 depletion partially rescued the programmed cell death phenotype observed. The 9-gene group includes *KIF14*, a known oncogene associated with aggressive tumor qualities in many cancers, linking HDAC11 epigenetic regulation to the hallmarks of aggressive cancers. Taken together, our data illustrate an important role for HDAC11 in cell cycle progression and viability of *MYCN*-amplified neuroblastoma cells, and illuminates HDAC11 as a potential novel drug target for this subgroup of high-risk neuroblastomas.

## Materials and methods

### Cell culture and chemicals

The BE(2)-C and SK-N-AS neuroblastoma cell lines were obtained from ECACC (Salisbury, UK), and the IMR-32 and SH-SY5Y cell lines from the DSMZ (Braunschweig, Germany). Cell lines were monitored for infections by high-throughput multiplex cell contamination testing.^75^ Cell line authenticity was validated by high-throughput SNP-based assays.^76^ BE(2)-C, SK-N-AS, IMR-32 and SK-N-AS cell lines were cultured in DMEM (Lonza, Basel, Switzerland) supplemented with 10% FCS (Sigma-Aldrich, Hamburg, Germany) and 1% non-essential amino acids (NEAA; Lonza) at 37°C and 5% CO_2_. The broad range caspase inhibitor, zVAD.fmk (Bachem, Heidelberg, Germany), was directly added to the cell culture medium to obtain a final concentration of 20 μM, whereas controls were treated with DMSO.

### Microscopy

Living native cells were examined with an inverted widefield microscope (Olympus CKX41, Tokyo, Japan). Software Cell^B (Olympus) was used for the acquisition of microscopic images. Fixed, DAPI-labeled cells were viewed under both the Olympus CKX41 and a laser-scanning confocal microscope (Zeiss LSM700, Oberkochen, Germany) equipped with the ZEN 2012 blue edition software (Zeiss). Operators performing the quantification of DAPI-labeled mitotic cells were blinded to the treatment group. Positive cells were counted using the Cell^B software (Olympus) counting tool.

### Transfection of siRNAs and DNA plasmids

For knockdown experiments, cells were transiently transfected with 25 nM siRNA (Supplementary Table S1) using the HiPerFect method (Qiagen, Hilden, Germany) according to the manufacturer's directions. For plasmid transfection, the Effectene method (Qiagen) was used according to the manufacturer's manual. Applying the GATEWAY technology (Invitrogen, Carlsbad, CA, USA), the *RACGAP1* complementary DNA (cDNA) sequence (EU176264) was cloned from the pENTR221 vector into the destination vector pT-REx-DEST30. The empty pT-REx-DEST30 vector and the LacZ expression vector, pT-REx/GW30/LacZ (Invitrogen), served as controls. For the expression of Myc-tagged HDAC11, the MYC tag sequence (MEQKLISEEDL) was N-terminally inserted into the expression vector pcDNA3.1 carrying the wild-type *HDAC11* sequence.^19^ The correct sequences of all inserts were verified by DNA sequencing (GATC, Konstanz, Germany).

### RNA extraction, cDNA synthesis and qRT-PCR

Total RNA was isolated from BE(2)-C and IMR-32 cell lines using the RNeasy Mini Kit (Qiagen). The Thermo Scientific First-Strand cDNA Synthesis Kit (Thermo Scientific, Waltham, MA, USA) was used to transcribe cDNAs for qRT-PCR analysis. Relative gene expression was measured using SYBR Green Dye (Eurogentec, Cologne, Germany) on an ABI Prism 7500 thermal cycler (Perkin-Elmer Applied Biosystems, Weiterstadt, Germany). All primers used in qRT-PCR are listed in the Supplementary Table S2. Data were analyzed using Applied Biosystems 7500 software v2.0.5 (Thermo Scientific), and changes in gene expression were calculated using the ΔΔ*C*_t_ method.

### Western blotting

Western blots were performed as described^19, 77^ using the following antibodies: mouse monoclonal anti-*β*-actin (clone AC-15, Sigma-Aldrich, St. Louis, MO, USA), rabbit polyclonal anti-CASP3 (Cell Signaling, Danvers, MA, USA), mouse monoclonal anti-GAPDH (clone 6C5; Merck Millipore, Darmstadt, Germany), mouse monoclonal anti-HDAC1 (clone 10E2; Abcam, Cambridge, UK), mouse monoclonal anti-HDAC2 (clone 3F3; Abcam), rabbit polyclonal anti-HDAC3 (clone H-99; Santa Cruz Biotechnology, Dallas, TX, USA), rabbit polyclonal anti-HDAC8 (Abcam), mouse monoclonal anti-MYC tag (GeneTex, Irvine, CA, USA), mouse monoclonal anti-PARP (Cell Signaling), mouse monoclonal anti-phospho H3 (Ser10) (Cell Signaling), rabbit monoclonal anti-histone H3 (Cell Signaling) and rabbit polyclonal anti-RACGAP1 (Santa Cruz Biotechnology). Band density was analyzed using ImageJ 1.47p software (Wayne Rasband, National Institute of Health, Bethesda, MD, USA) on western blots, and results were normalized to the respective loading controls.

### Trypan blue exclusion and CellTiter-Glo assays

Cell number and viability were semi-automatically measured with the VI-CELL Cell Viability Analyzer (Beckman, Krefeld, Germany) based on the trypan blue exclusion method.^20^ The CellTiter-Glo luminescent cell viability assay (Promega, Fitchburg, WI, USA) was used according to the manufacturer's directions to measure the amount of intracellular ATP.

### Flow cytometry

Cell cycle distribution and DNA fragmentation was determined on a FACS Calibur flow cytometer (BD Biosciences, Heidelberg, Germany) using the CellQuest Pro software (BD Biosciences) after cell nuclei were stained with propidium iodide.^19^ The FITC Annexin V Apoptosis Detection Kit I (BD Pharmingen, Heidelberg, Germany) was used to quantify phosphatidylserine at the outer cell membrane.

### Caspase activity assay

Cells were seeded, treated as indicated, collected with supernatant and lysed in cell lysis buffer (Biovision, Mountain View, CA, USA) for 10 min on ice. Protein concentrations were normalized using the Pierce assay. Thereafter, reaction buffer (MBL International, Woburn, MA, USA) and AFC-labeled caspase 3-specific peptide, DEVD (Biomol, Hamburg, Germany), were added. Caspase 3-like activity was measured at 37 °C in black 96-well plates using a fluorescence plate reader with a 380 nm excitation filter and a 530 nm emission filter.^19^ The Caspase Glo 3/7 assay (Promega) facilitating direct measurements without cell harvest and lysis was used for medium-throughput analyses, and results were normalized to the amount of intracellular ATP measured by the CellTiter-Glo assay.

### Probe labeling, Illumina Sentrix BeadChip array hybridization and microarray scanning

RNA isolated from cell lines as described above was resuspended in TE buffer and eluted in water. Quality of total RNA was checked by gel analysis using the total RNA NanoChip Assay on an Agilent 2100 Bioanalyzer (Agilent Technologies GmbH, Berlin, Germany). Samples with RNA indices >8.5 were selected for expression profiling. RNA concentrations were determined with a NanoDrop spectrophotometer (NanoDrop Technologies, Wilmington, DE, USA). Biotin-labeled cRNA samples were prepared for hybridization on Illumina Human Sentrix-12 BeadChip arrays (Illumina Inc., San Diego, CA, USA) according to the sample labeling procedure recommended by Illumina with minor modifications.^78^ In brief, 250 ng total RNA was used for cDNA synthesis, followed by an amplification and labeling step to synthesize biotin-labeled cRNA according to the Illumina TotalPrep RNA Amplification Kit (Life Technologies, Carlsbad, CA, USA). The cRNA was column-purified with the TotalPrep RNA Amplification Kit and eluted in 60 *μ*l of water. The cRNA quality was controlled with the RNA NanoChip Assay on an Agilent 2100 Bioanalyzer, and the cRNA content was spectrophotometrically quantified with a NanoDrop.

Hybridization was performed at 58°C in GEX-HCB buffer (Illumina Inc.) at a concentration of 100 ng cRNA/*μ*l, unsealed in a wet chamber for 20 h. Thereafter, mismatch control oligonucleotides, biotinylation control oligonucleotides and spike-in controls for low-, medium- and highly abundant RNAs were added. Microarrays were washed once in High Temp Wash buffer (Illumina Inc.) at 55°C and twice in E1BC buffer (Illumina Inc.) at room temperature for 5 min. In between, microarrays were washed with ethanol at room temperature. After blocking for 5 min in 4 ml of 1% (w/v) Blocker Casein in phosphate-buffered saline Hammarsten grade (Pierce Biotechnology Inc., Rockford, IL, USA), array signals were developed by a 10 min incubation in 2 ml of 1 *μ*g/ml Cy3-streptavidin solution (Amersham Biosciences, Buckinghamshire, UK) and 1% blocking solution. After a final wash in E1BC, microarrays were dried and scanned using an iScan array scanner. Data extraction was done for all beads individually. Outliers, identified as having >2.5 MAD (median absolute deviation), were removed. All remaining data points were used for the calculation of the mean average signal and S.D. for a given probe. The data discussed in this publication have been deposited in NCBI's Gene Expression Omnibus^79^ and are accessible through GEO Series accession number GSE77080 (http://www.ncbi.nlm.nih.gov/geo/query/acc.cgi?acc=GSE77080).

### Statistical analysis

Microarray data obtained in duplicates were normalized using quantile normalization in the 'R programming language'. Quantile-normalized Illumina mRNA data were log_2_ transformed. Differentially expressed transcripts were identified for each *HDAC11* siRNA separately in comparison to the negative control transfection using the empirical Bayes approach as implemented in the Bioconductor package limma.^80^ Time points were tested globally using moderated F-statistics and individually using moderated *t*-statistics, both based on the same linear model. All *P*-values were adjusted for multiple testing using the Benjamini–Hochberg correction.^81^ Next, transcripts that showed a significant regulation at two or more time points in the same direction and no significant regulation in the opposite direction, were selected. The overlap of these selected transcripts from both siRNAs defined the primary list of regulated transcripts (hits). This analysis was performed separately for each cell line. GO terms were analyzed for over-representation and enrichment. First, hypergeometric tests were used to test for over-representation of GO terms within the hit list. Second, a gene set enrichment analysis was performed using the minimal F-statistics from both siRNA models as a global measurement of regulation. Taking the minimal moderated F-statistic value from both linear models can be considered a conservative approach because the lesser strength of regulation from both siRNAs is selected to represent the transcript, thus mimicking the requirement to show regulation in both siRNAs. In case multiple transcripts mapped to the same Entrez Gene ID, duplicates were removed for both analyses by using only the transcript that showed the strongest regulation to represent the gene. Again, *P*-values were adjusted for multiple testing using Benjamini–Hochberg correction.^81^ GO analyses were carried out for each cell line separately with the Bioconductor package HTSanalyzeR.^82^ GO terms showing both a significant over-representation and enrichment were selected for further investigation. All analyses were carried out using R,^83^ and all tests were two-sided.

Effects of HDAC11 depletion on phenotype compared to those of negative control siRNA transfection were analyzed by a mixed linear model with a fixed effect for HDAC11-depleted samples in comparison to negative control treated cultures and random intercept for each individual *HDAC11* or negative control siRNA using SAS PROC MIXED, SAS Version 9.2 (SAS Institute Inc., Cary, NC, USA). Comparison of qRT-PCR data was performed with a paired two-tailed *t*-test (GraphPad Prism version 5.01, GraphPad Software, Inc., La Jolla, CA, USA). *P*-values below 0.05 were considered statistically significant.

## Figures and Tables

**Table 1:** Correlation of mRNA expression of HDAC11-regulated genes with patient outcome in the 476 neuroblastoma cohort by Oberthuer *et al.*^30^

	**Overall survival/entire cohort**				**Event-free survival/entire cohort**			
**Gene**	**Cutoff**	***n* (high)**	***n* (low)**	***P*-value**^a^	**Cutoff**	***n* (high)**	***n* (low)**	***P*-value**^a^
*CCNE1*	4463.4	80	396	8.4 E^−22^	4416.3	82	394	1.7 E^−13^
*CENPA*	1239.5	136	340	5.0 E^−24^	1146.9	146	330	4.2 E^−^^22^
*CENPE*	2518.9	133	343	2.0 E^−22^	2258.1	155	321	1.7 E^−21^
*DLGAP5*	2443.4	142	334	6.8 E^−^^21^	2158.5	160	316	2.2 E^−19^
*KIF14*	293.0	169	307	1.3 E^−^^15^	293.0	169	307	5.2 E^−14^
*KIF23*	4447.3	93	383	8.3 E^−^^14^	4447.3	93	383	4.4 E^−12^
*MAD2L1*	6671.7	163	313	1.5 E^−22^	6671.7	163	313	1.0 E^−^^20^
*RACGAP1*	12864.4	63	413	2.0 E^−09^	12949.5	61	415	1.2 E^−^^11^
*UHRF1*	2461.8	151	325	2.1 E^−27^	2211.7	171	305	8.1 E^−^^20^
								
	**Overall survival/MYCN single-copy tumors**				**Event-free survival/MYCN single-copy tumors**			
**Gene**	**Cutoff**	***n*(high)**	***n*(low)**	***P*-value**^a^	**Cutoff**	***n*(high)**	***n*(low)**	***P*-value**^a^
*CCNE1*	4463.4	40	365	2.5 E^−^^07^	4815.1	28	377	1.4 E^−06^
*CENPA*	1671.9	51	354	9.3 E^−^^11^	1146.9	87	318	1.7 E^−13^
*CENPE*	2645.6	73	332	1.1 E^−^^11^	2258.1	97	308	8.0 E^−^^16^
*DLGAP5*	2158.5	104	301	7.9 E^−11^	2158.5	104	301	4.7 E^−^^13^
*KIF14*	617.3	23	382	4.2 E^−09^	293.0	118	287	3.4 E^−^^06^
*KIF23*	4447.3	58	347	3.4 E^−^^06^	2949.5	138	267	2.7 E^−^^07^
*MAD2L1*	6898.8	97	308	8.7 E^−^^14^	6696.5	100	305	9.5 E^−^^15^
*RACGAP1*	15662.4	17	388	1.4 E^−^^07^	12949.5	45	360	5.1 E^−08^
*UHRF1*	2461.8	92	313	1.7 E^−15^	2023.2	129	276	2.8 E^−14^

*P*-values were adjusted for multiple testing due to cut-point search according to Lausen^84^

a Higher gene expression was always associated with worse prognosis
