# Supplementary material for: Neuroblastoma cells depend on HDAC11 for mitotic cell cycle progression and survival
Source: Cell Death Dis. 2017 Mar 2;8(3):e2635–. doi: 10.1038/cddis.2017.49 (PMC5386552; doi:10.1038/cddis.2017.49)
Supplement: Supplementary Information [file cddis201749x1.docx]

**SUPPLEMENTARY INFORMATION**

**Neuroblastoma cells depend on HDAC11**

**for mitotic cell cycle progression and survival**

Theresa M. Thole, Marco Lodrini, Johannes Fabian, Jasmin Wünschel, Sebastian Pfeil,

Thomas Hielscher, Annette Kopp-Schneider, Ulrike Heinicke, Simone Fulda,

Olaf Witt, Angelika Eggert, Matthias Fischer and Hedwig E. Deubzer

**Table of Contents**

| **Supplementary Materials & Methods** | |  | **starting p.2** |
| --- | --- | --- | --- |
| **Supplementary Tables** | |  | **starting p.3** |
| **Supplementary Figure Legends** | |  | **starting p.6** |
| **Supplementary Figure S1** | **Related to Figures 1-2** | |  |
| **Supplementary Figure S2** | **Related to Figure 1** | |  |
| **Supplementary Figure S3** | **Related to Figure 1** | |  |
| **Supplementary Figure S4** | **Related to Figure 2** | |  |
| **Supplementary Figure S5** | **Related to Table 1** | |  |
| **Supplementary Figure S6** | **Related to Figure 5** | |  |
| **Supplementary References** | |  | **starting p.7** |

**Supplementary Materials and Methods**

**Characteristics of patients and tumor samples**

*CCNE1*, *CENPA*, *CENPE*, *DLGAP5*, *KIF14*, *KIF23*, *MAD2L1*, *RACGAP1* and *UHRF1* mRNA expression values were derived from existing whole-genome expression profiles of 476 primary neuroblastoma samples.^1^ Tumor samples were collected internationally between 1989 and 2007 and had at least 60% tumor cell count. A second independent dataset was derived from publicly accessible whole-genome expression profiles of 88 neuroblastomas^2^ via the R2 microarray analysis and visualization platform (<http://r2.amc.nl>). Probesets with the highest average signal for each gene were selected for analysis.

**Statistical analysis**

Kaplan-Meier curves relating *CCNE1*, *CENPA*, *CENPE*, *DLGAP5*, *KIF14*, *KIF23*, *MAD2L1*, *RACGAP1* and *UHRF1* expression to patient survival in the 476 neuroblastoma cohort^1^ were plotted using the Survival package for the R programming language. The optimal cut-off for overall and event-free survival analyses was chosen as the expression value where the log-rank statistic for the separation of both survival curves reached a maximum. This value was calculated using the Maxstat package for the R programming language. Reported *P* values were calculated using the *P* value approximation method according to Lausen.^3^ Kaplan-Meier analyses for the 88 neuroblastoma cohort^3^ were performed online in the R2 platform (<http://r2.amc.nl>), and the resulting survival curves and *P* values (log-rank test) were downloaded. A Pearson analysis^4^ was performed to test the correlation among the mRNA expression values of the nine selected genes in the 476 neuroblastoma cohort^1^. The regression model according to Goeman and colleagues^5^ was used to test whether high-level mRNA expression of these nine genes correlates with amplified (n=67) versus single copy *MYCN* status (n=77) in the 144 high-risk neuroblastomas of the Oberthuer cohort^1^ as defined by the International Neuroblastoma Risk Group (INRG) staging system^6^.

**Supplementary Table S1.** Small interfering RNAs used for transient RNA interference.

| **gene** | **siRNA abbreviation** | **target sequence (5’-3’)** | **company** | **ID#** |
| --- | --- | --- | --- | --- |
| *CCNE1* | no. 1  no. 2 | CAG GGT ATC AGT GGT GCG ACA  ACC GGG TTT ACC CAA ACT CAA | Qiagen  Qiagen | SI00024024  SI02625693 |
| *CENPA* | no. 1  no. 2 | CAC CGT TCC AAA GGC CTG AAA  CAG AGC CAT GAC TAG ATC CAA | Qiagen  Qiagen | SI04138491  SI04230177 |
| *CENPE* | no. 1  no. 2 | CAG GTT AAT CCT ACC ACA CAA  TAA GAT GGT CCT TGA GGA GAA | Qiagen  Qiagen | SI02653217  SI04434696 |
| *DLGAP5* | no. 1  no. 2 | AACGAGAGTGATGTTCGAGCA  TGGGTCGTTATAGACCTGATA | Qiagen  Qiagen | SI04438378  SI04438385 |
| *HDAC1* | no. 1  no. 2 | CAC CCG GAG GAA AGT CTG TTA  in exons 7 and 8 | Qiagen  Ambion | SI02663472  120418 |
| *HDAC2* | no. 1  no. 2 | ACG GTC AAT AAG ACC AGA TAA  CTG GGT TGT TTC AAT CTA ACA | Qiagen  Qiagen | SI00434952  SI00434959 |
| *HDAC3* | no. 1  no. 2 | GAC CAT GAC AAT GAC AAG GAA  CAC CCG CAT CGA GAA TCA GAA | Qiagen  Qiagen | SI00057337  SI03057901 |
| *HDAC8* | no. 1  no. 2 | in exons 1 and 2  in exon 5 | Ambion  Ambion | 120597  2120599 |
| *HDAC11* | no. 1  no. 2 | in exon 8  GCG AGA CTT CAT GGA CGA CAA | Ambion  Qiagen | 130749  SI03105186 |
| *KIF14* | no. 1  no. 2 | ATG GTT AAT CGT GCT CCA GAA  CAG ACA TGA TAT ATC AGA TAA | Qiagen  Qiagen | SI02780876  SI02781163 |
| *KIF23* | no. 1  no. 2 | CAG AAT AAA CTC TGG GTT AAA  AAG GCT GAA GAT TAT GAA GAA | Qiagen  Qiagen | SI00069349  SI02653483 |
| *MAD2L1* | no. 1  no. 2 | TCC GCC TTC GTT CAT TTA CTA  CTG AAA GTA ACT CAT AAT CTA | Qiagen  Qiagen | SI00036666  SI02653847 |
| *RACGAP1* | no. 1  no. 2 | CAG GTG GAT GTA GAG ATC AAA  AAG GAT TAG ACC AGA GGA TTT | Qiagen  Qiagen | SI00101178  SI04954873 |
| *UHRF1* | no. 1  no. 2 | CCA CGT GAA ATA CGA CGA CTA  CCG CTA CGA TGG CAT CTA CAA | Qiagen  Qiagen | SI03074680  SI03081337 |
| Neg Co | no. 1 |  | Qiagen | SI03650318 |
| Neg Co | no. 2 |  | Thermo Scientific | D-001220-01-20 |

**Supplementary Table S2.** Primers used in qRT-PCR.

| **gene** | **forward primer (5’-3’)** | **reverse primer (5’-3’)** | **Qiagen ID#** | **reference** |
| --- | --- | --- | --- | --- |
| *ACTB* | GCA TCC CCC AAA GTT CAC AA | AGG ACT GGG CCA TTC TCC TT |  | 7 |
| *CCNE1* |  |  | QT00041986 | - |
| *CENPA* |  |  | QT00001057 | - |
| *CENPE* |  |  | QT00013006 | - |
| *DLGAP5* |  |  | QT00013545 | - |
| *HDAC1* | TGA CGA GTC CTA TGA GGC CAT T | CCG CAC TAG GCT GGA ACA TC |  | 8 |
| *HDAC2* | TGT GAG ATT CCC AAT GAG TTG C | GGT AAC ATG CGC AAA TTT TCA A |  | 8 |
| *HDAC3* | CCT CAC TGA CCG GGT CAT G | ACC TGT GCC AGG GAA GAA GTA A |  | 8 |
| *HDAC8* | CCA AGA GGG CGA TGA  TGA TC | GTG GCT GGG CAG TCA  TAA CC |  | 8 |
| *HDAC11* | CAA TGG GCA TGA GCG AGA C | TGT GGC GGT TGT AGA CAT CC |  | 9 |
| *HPRT1* | TGA CAC TGG CAA AAC AAT GCA | GGT CCT TTT CAC CAG CAA GCT |  | 10 |
| *KIF14* |  |  | QT00001827 | - |
| *KIF23* |  |  | QT00048419 | - |
| *MAD2L1* |  |  | QT00094955 | - |
| *NASP* |  |  | QT00048832 | - |
| *RACGAP1* |  |  | QT00085953 | - |
| *SDHA* | TGG GAA CAA GAG GGC ATC TG | CCA CCA CTG CAT CAA ATT CAT G |  | 10 |
| *UHRF1* |  |  | QT00018256 | - |
| *18-S* rRNA | GAG GAT GAG GTG GAA CGT GT | TCT TCA GTC GCT CCA GGT CT |  | - |

**Supplementary Table S3.** Correlation of mRNA expression of HDAC11-regulated genes with patient outcome in the 88 neuroblastoma cohort by Molenaar and colleagues^2^.

|  | **Overall** **survival** | | | | **Event-free** **survival** | | | |
| --- | --- | --- | --- | --- | --- | --- | --- | --- |
| **Gene** | **Cut-off** | ***n* (high)** | ***n* (low)** | ***P*-value^*^** | **Cut-off** | ***n* (high)** | ***n* (low)** | ***P*-value^*^** |
| *CCNE1* | 79.7 | 19 | 69 | 6.7 E^-04^ | 79.7 | 19 | 69 | 0.041 |
| *CENPA* | 94.5 | 20 | 68 | 3.7 E^-4^ | 90.4 | 23 | 65 | 4.8 E^-06^ |
| *CENPE* | 77.1 | 60 | 28 | 5.1 E^-03^ | 77.1 | 60 | 28 | 6.6 E^-03^ |
| *DLGAP5* | 157.8 | 33 | 55 | 6.2 E^-05^ | 110.0 | 54 | 34 | 5.8 E^-05^ |
| *KIF14* | 229.2 | 33 | 55 | 4.8 E^-03^ | 177.5 | 49 | 39 | 2.3 E^-03^ |
| *KIF23* | 86.2 | 43 | 45 | 6.9 E^-04^ | 75.0 | 48 | 40 | 1.1 E^-04^ |
| *MAD2L1* | 609.1 | 49 | 39 | 7.8 E^-03^ | 609.1 | 49 | 39 | 0.019 |
| *RACGAP1* | 731.4 | 29 | 59 | 0.174 | 391.2 | 65 | 23 | 0.304 |
| *UHRF1* | 363.5 | 36 | 52 | 1.7 E^-05^ | 323.4 | 41 | 47 | 8.5 E^-04^ |

**^*^** Higher gene expression was always associated with worse prognosis. *P*-values were adjusted for multiple testing due to cut-point search using the Bonferroni method.

**SUPPLEMENTARY FIGRUE LEGENDS**

**Supplementary Figure S1.** Knockdown efficacy of siRNAs directed against *HDACs 1*, *2*, *3*, *8* and *11*. (**a-b**) *HDAC11* mRNA expression in BE(2)-C (**a**) and IMR-32 cells (**b**) following RNAi for 24-96 h (qRT-PCR; mean fold change to mock ± SD, n≥3). (**c-d**) MYC-tag HDAC11 protein expression (western blotting) in BE(2)-C (**c**) and IMR-32 cells (**d**) 72 h after MYC-tag HDAC11 plasmid and 48 h after siRNA transfection. (**e**-**h**) HDAC1, 2, 3 and 8 protein expression (western blotting) in BE(2)-C cells following RNAi for 54 h. β-actin served as loading control.

**Supplementary Figure S2.** HDAC11 depletion in *MYCN* single copy neuroblastoma cell lines causes minor effects. (**a**) *HDAC11* mRNA expression in SH-SY5Y and SK-N-AS cells following RNAi for 96 h (qRT-PCR; mean fold change to mock ± SD, n=3). (**b**) Intracellular adenosine triphosphate (ATP) content of SH-SY5Y and SK-N-AS cultures 96 h after transfection with *HDAC11*- or negative control siRNAs (mean fold change over mock transfected cells ± SD is shown, n=3). (**c**-**d**) Viable and dead SH-SY5Y and SK-N-AS cell count using a semi-automatic VI-CELL Cell Viability Analyzer 96 h after transfection with *HDAC11*- or negative control siRNAs. Mean fold change over viable mock transfected cells ± SD is shown in (**c**), n=3. Mean percentage of dead cells is shown in (**d**), n=3. ^*^*P*<0.05; ^***^*P*≤0.001.

**Supplementary Figure S3.** HDAC11 depletion causes a G2/M arrest in BE(2)-C cells. (**a**-**d**) BE(2)-C cells were transfected with a negative control siRNA or a siRNA directed against *HDAC11* for 24 – 96 h, stained with propidium iodide and analyzed for cell cycle distribution by flow cytometry. (**e**) Representative FACS histograms 48 h after HDAC11 depletion in BE(2)-C cells. ^***^*P*≤0.001.

**Supplementary Figure S4.** Representative FACS histograms. BE(2)-C and IMR-32 cells were transfected with negative control siRNA or two different siRNAs directed against *HDAC11* and stained at 72 h with Annexin V (**a**) or at 96 h with propidium iodide (**b**).

**Supplementary Figure S5.** Heatmap visualizing Pearson’s correlation coefficients among *CCNE1*, *CENPA*, *CENPE*, *DLGAP5*, *KIF14*, *KIF23*, *MAD2L1*, *RACGAP1* and *UHRF1* mRNA expression values in the 476 neuroblastoma tumor cohort^1^. No linear relationship between variables, r=0; perfect positive linear relationship between variables, r=1.

**Supplementary Figure S6.** RACGAP1 expression altered by RNAi and/or plasmid-mediated enforced expression. (**a**-**b**) RACGAP1 levels (western blotting) in BE(2)-C (**a**) and IMR-32 cells (**b**) 54 h after transient transfection of two different siRNAs directed against *HDAC11* or respective negative controls. (**c**) RACGAP1 levels (western blotting) in BE(2)-C cells 54 h after *HDAC11* siRNA transfection and 30 h after *RACGAP1* or *LacZ* plasmid transfection. β-actin served as loading control.

**SUPPLEMENTARY REFERENCES**

1. Oberthuer A, Hero B, Berthold F, Juraeva D, Faldum A, Kahlert Y*, et al.* Prognostic impact of gene expression-based classification for neuroblastoma. *J Clin Oncol* 2010; **28**: 3506-3515.

2. Molenaar JJ, Koster J, Zwijnenburg DA, van Sluis P, Valentijn LJ, van der Ploeg I*, et al.* Sequencing of neuroblastoma identifies chromothripsis and defects in neuritogenesis genes. *Nature* 2012; **483**: 589-593.

3. Lausen B, Schumacher M. Maximally selected rank statistics. *Biometrics* 1992; **48**: 85.

4. Gayen AK. The frequency distribution of the product-moment correlation coefficient in random samples of any size drawn from non-normal universes. *Biometrika* 1951; **38**: 219-247.

5. Goeman JJ, van de Geer SA, de Kort F, van Houwelingen HC. A global test for groups of genes: testing association with a clinical outcome. *Bioinformatics* 2004; **20**: 93-99.

6. Cohn SL, Pearson AD, London WB, Monclair T, Ambros PF, Brodeur GM*, et al.* The International Neuroblastoma Risk Group (INRG) classification system: an INRG Task Force report. *J Clin Oncol* 2009; **27:** 289-297.

7. Deubzer HE, Ehemann V, Westermann F, Heinrich R, Mechtersheimer G, Kulozik AE*, et al.* Histone deacetylase inhibitor Helminthosporium carbonum (HC)-toxin suppresses the malignant phenotype of neuroblastoma cells. *Inter J Cancer* 2008; **122**: 1891-1900.

8. Oehme I, Deubzer HE, Wegener D, Pickert D, Linke JP, Hero B*, et al.* Histone deacetylase 8 in neuroblastoma tumorigenesis. *Clin Cancer Res* 2009; **15**: 91-99.

9. Gao L, Cueto MA, Asselbergs F, Atadja P. Cloning and functional characterization of HDAC11, a novel member of the human histone deacetylase family. *J Biol Chem* 2002; **277**: 25748-25755.

10. Fischer M, Skowron M, Berthold F. Reliable transcript quantification by real-time reverse transcriptase-polymerase chain reaction in primary neuroblastoma using normalization to averaged expression levels of the control genes HPRT1 and SDHA. *J Mol Diagn* 2005; **7**: 89-96.
