# Supplementary figures and images for: Neuroblastoma cells depend on HDAC11 for mitotic cell cycle progression and survival
Source: Cell Death Dis. 2017 Mar 2;8(3):e2635–. doi: 10.1038/cddis.2017.49 (PMC5386552; doi:10.1038/cddis.2017.49)

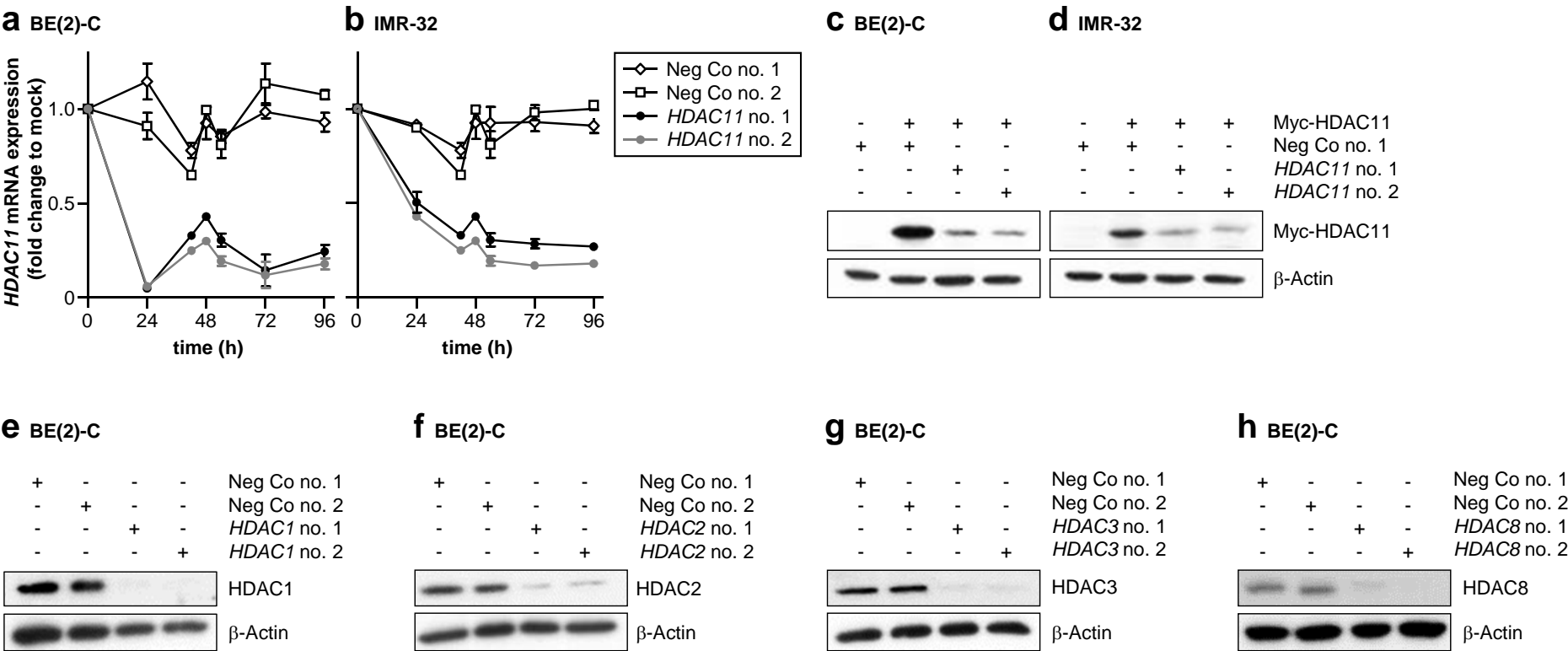

Supplement: Supplementary Figure 1 [file cddis201749x2.pdf]

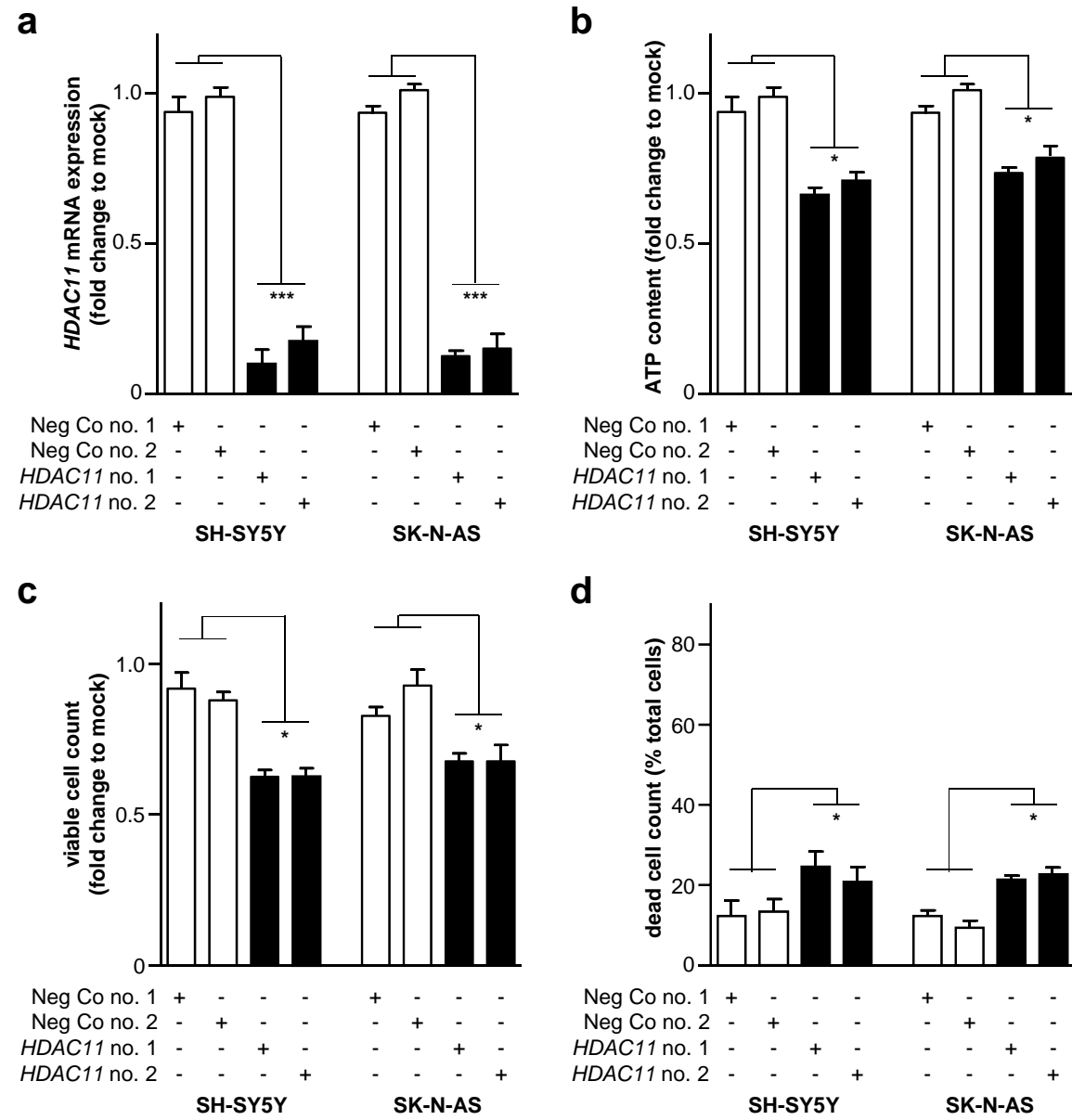

Supplement: Supplementary Figure 2 [file cddis201749x3.pdf]

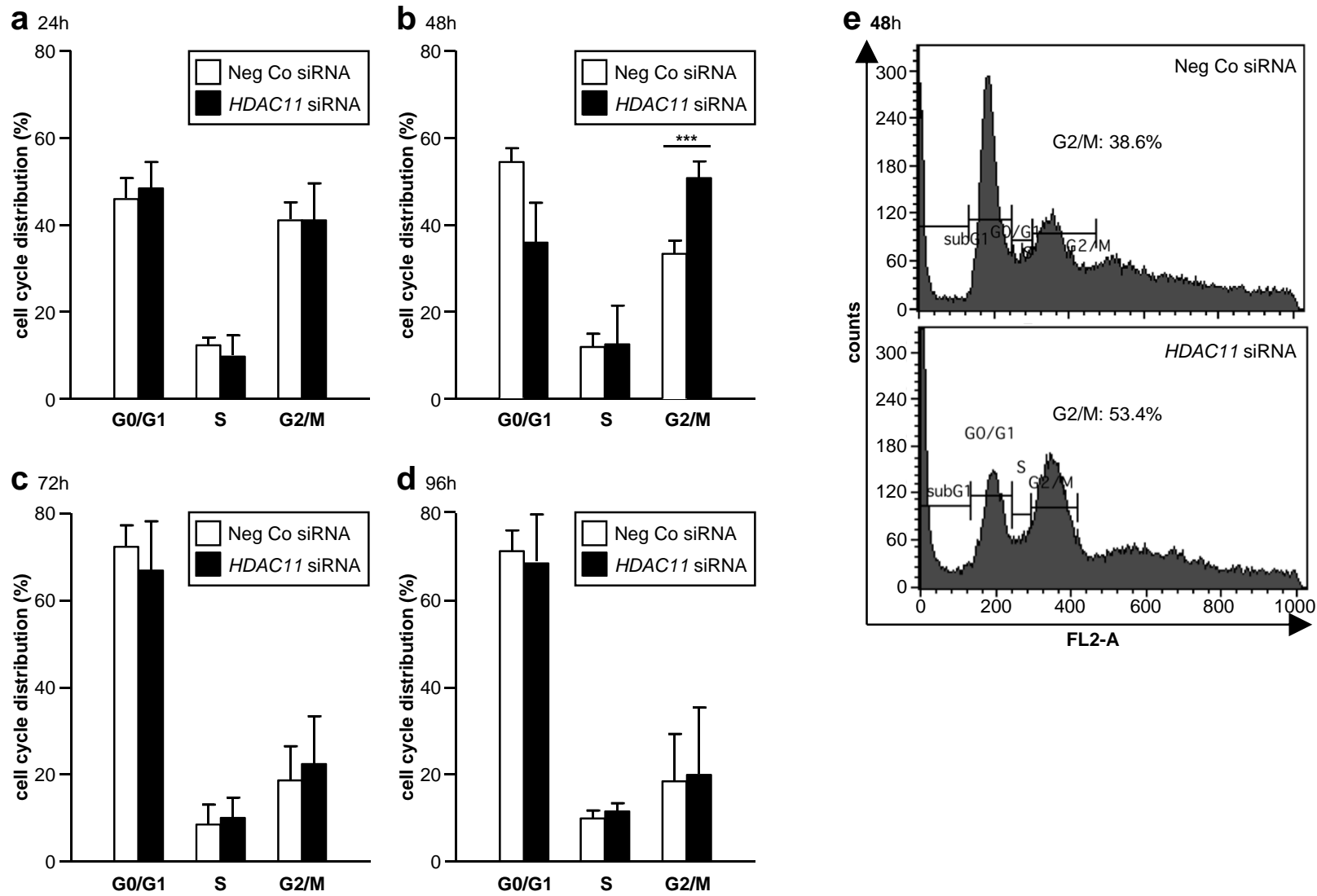

Supplement: Supplementary Figure 3 [file cddis201749x4.pdf]

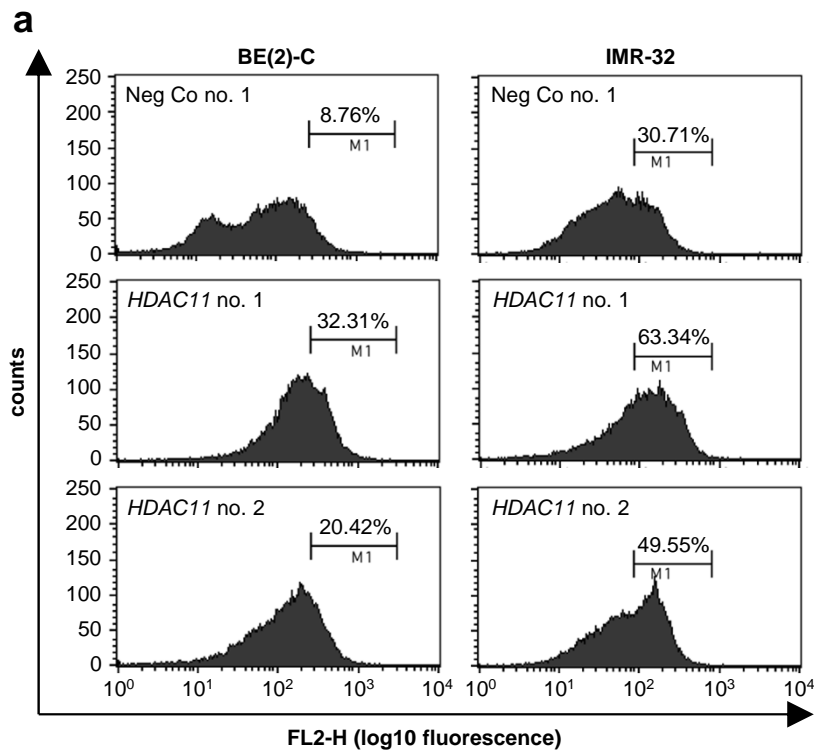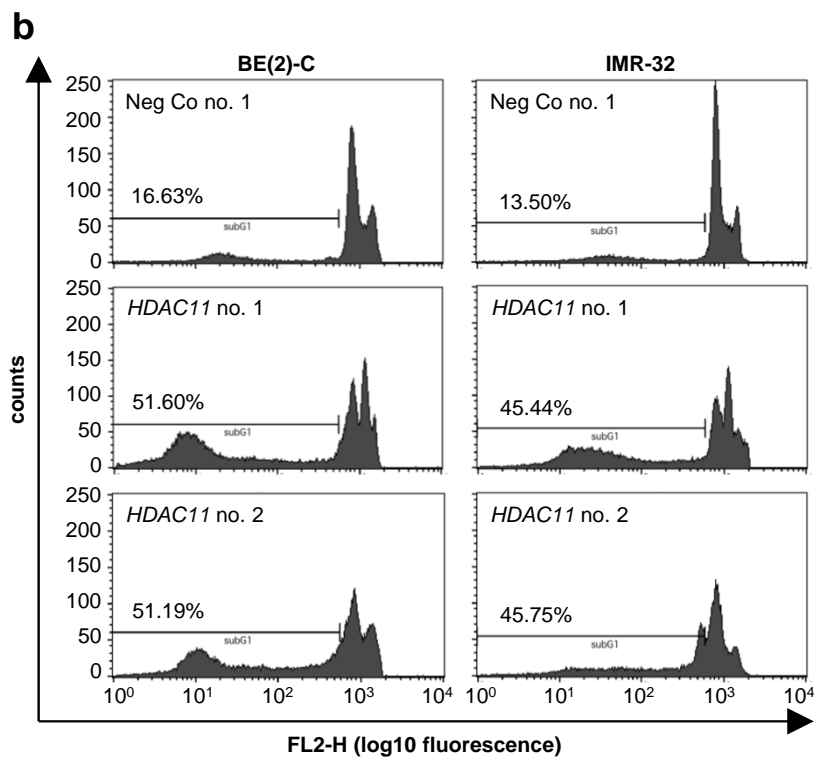

Supplement: Supplementary Figure 4 [file cddis201749x5.pdf]

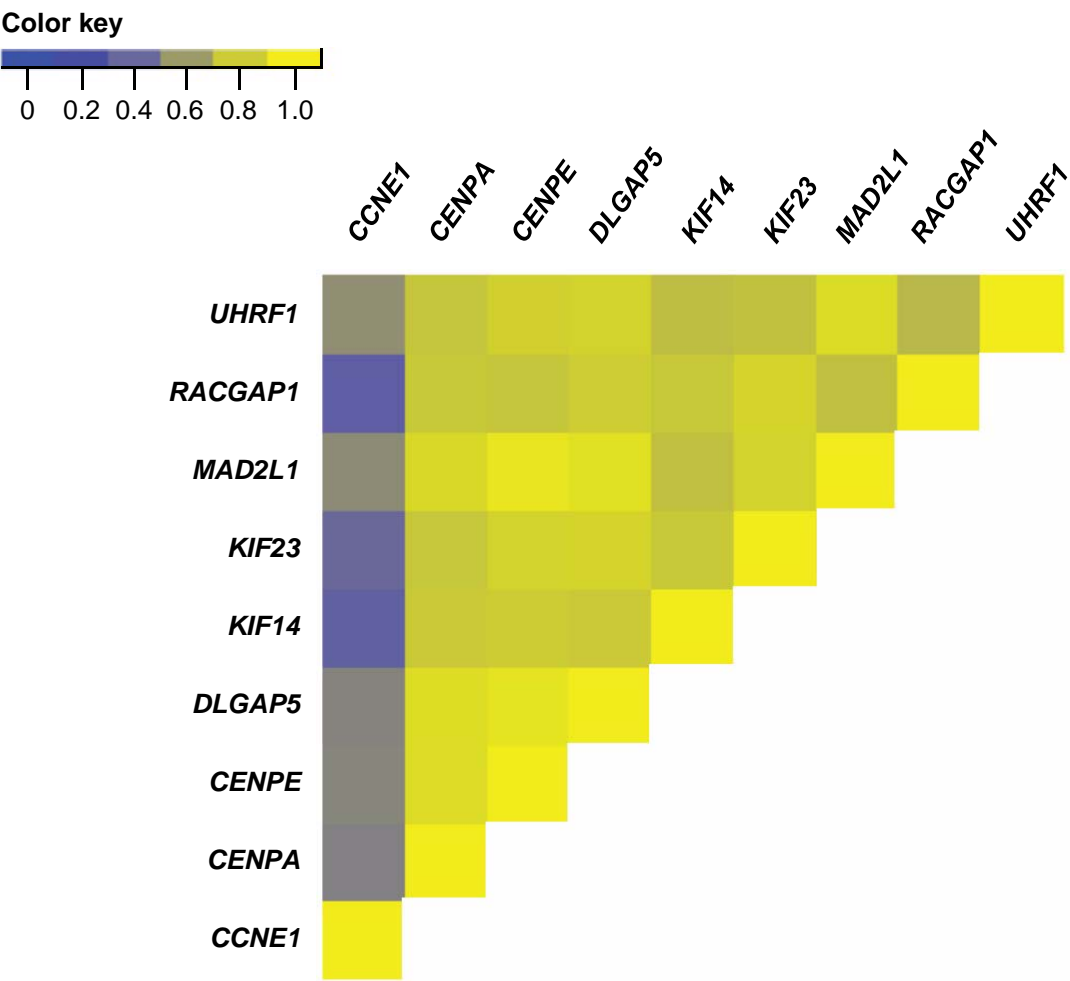

Supplement: Supplementary Figure 5 [file cddis201749x6.pdf]

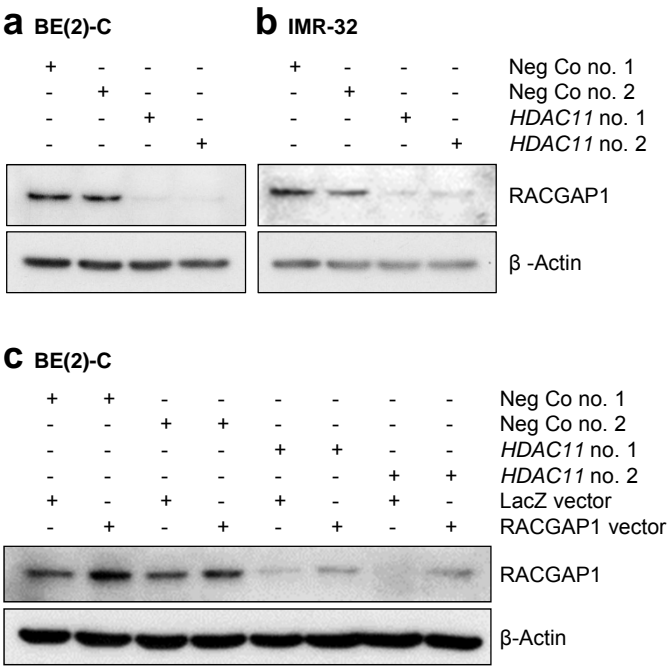

Supplement: Supplementary Figure 6 [file cddis201749x7.pdf]
